# A Comparison of FPGA and GPGPU Designs for Bayesian Occupancy Filters

**DOI:** 10.3390/s17112599

**Published:** 2017-11-11

**Authors:** Luis Medina, Miguel Diez-Ochoa, Raul Correal, Sergio Cuenca-Asensi, Alejandro Serrano, Jorge Godoy, Antonio Martínez-Álvarez, Jorge Villagra

**Affiliations:** 1 University Institute for Computing Research, University of Alicante, 03690 San Vicente del Raspeig, Spain; lmedina@dtic.ua.es (L.M.); sergio@dtic.ua.es (S.C.-A.); aserrano@dtic.ua.es (A.S.); amartinez@dtic.ua.es (A.M.-Á.); 2 Ixion Industry & Aerospace SL, Julian Camarilo 21B, 28037 Madrid, Spain; mdiezochoa@ixion.es (M.D.-O.); rcorreal@ixion.es (R.C.); 3 Centre for Automation and Robotics (UPM-CSIC), 28500 Arganda del Rey, Spain; jorge.godoy@csic.es

**Keywords:** Bayesian occupancy filter, FPGA, GPGPU, embedded system, ADAS

## Abstract

Grid-based perception techniques in the automotive sector based on fusing information from different sensors and their robust perceptions of the environment are proliferating in the industry. However, one of the main drawbacks of these techniques is the traditionally prohibitive, high computing performance that is required for embedded automotive systems. In this work, the capabilities of new computing architectures that embed these algorithms are assessed in a real car. The paper compares two ad hoc optimized designs of the Bayesian Occupancy Filter; one for General Purpose Graphics Processing Unit (GPGPU) and the other for Field-Programmable Gate Array (FPGA). The resulting implementations are compared in terms of development effort, accuracy and performance, using datasets from a realistic simulator and from a real automated vehicle.

## 1. Introduction

Intelligent vehicle technology is advancing at a vertiginous pace. However, the complexity of some highly uncertain and dynamic urban driving scenarios still hampers the deployment of fully automated vehicles. One of the most important challenges in those scenarios is the accurate perception of static and moving objects, to properly understand the spatio-temporal relationship between the subject vehicle and the relevant entities.

In well structured driving environments, such as highways, the types of static and dynamic objects are easily modeled and tracked using geometrical models and their parameters. However, urban driving scenarios are so heterogeneous and unpredictable that they are extremely complex to manage under a feature-based perception paradigm. In addition, the associated tracking methodology raises the classic problem of object association and state estimation, which are highly coupled.

Occupancy grids [1] overcome these difficulties, and in particular the Bayesian Occupancy Filter (BOF) [2] by projecting objects onto a compact regularly subdivided probabilistic grid, and tracking them as a set of occupied or dynamic cells. Under this paradigm, there is no need for higher level object models, resulting in: (i) a much higher insensitivity to the extreme variability of objects; and, (ii) avoidance of the association problem. In addition, it produces a compact representation model, as empty space is also conveniently represented for proper situational awareness.

The main drawback of this approach is its computational cost, unaffordable for automotive embedded systems [3]. Currently, common Electronic Control Unit (ECU), based on single or multicore microcontrollers (MCUs) [4] are used for low to medium-speed sensor data processing. The overall performance of these systems depends on the number of cores, their operating frequencies, memory bandwidth, and size. However, they are in general insufficient for processing high-demand algorithms in parallel with other data. As a result, MCU resource sharing (data acquisition, memory bus limitations, number of cores, frequency limitations etc.) to fuse data from high bandwidth sensors is prohibitive and the data are frequently fed into an occupancy grid that models the environment.

Likewise, current ECUs are dedicated embedded components, each one subject to a series of functional and safety requirements. As a result, state-of-the art vehicles can carry up to 100 ECUs on board using 5 km cable lengths. This situation creates a significant series of disadvantages, such as material costs, communication bandwidth limitations, latency problems, higher power consumption, potential robustness issues and high development and maintenance costs.

The integration of high-performance computing units are needed to overcome current limitations, fed by multiple sensor technologies such as radar, computer vision systems, LiDARs (Light Detection and Ranging) or even ultrasounds, complementing and reinforcing each other, and communicating over a reliable high-bandwidth network. Optimal usage of a huge amount of heterogeneous information requires the use of novel computing platforms and powerful algorithms that exploit their architecture as best they can.

Heterogeneous System on Chip (SoC) platforms can efficiently use different sensors for multiple functions and interconnect them with other systems—e.g., a camera is used for object detection and recognition, localization, and lane estimation. Additionally, parallel-processing resources offered by FPGA and GPGPU can be exploited to implement highly parallelizable computations. As a result, different functions, which, when available, are currently implemented in separate ECUs, can potentially be integrated within a single domain of a high-performance computing unit.

An in-depth study in this paper of the parallelization opportunities of new heterogeneous SoCs addresses the specific problem of object detection and tracking using the BOF paradigm. Although some previous works have optimized BOF designs for GPGPU platforms, in this paper, to the best of our knowledge, we are presenting the first implementation of the BOF using FPGA. In addition, a thorough comparison of BOF between heterogeneous SoCs using GPGPU (Nvidia Tegra K1) and FPGA (Xylinx Zynq) was carried out, using both synthetic sensor data from simulated and experimental datasets gathered by a real automated vehicle on open roads.

The remainder of this paper is as follows. Section 2 introduces and analyzes previous work on embedded systems for Advanced Driver Assistance Systems (ADAS), particularly those using multiple range sensors for object detection, world modeling and driving situation understanding. Then, a brief summary of the BOF is presented in Section 3, serving as the foundation for a description of the problem and the methodology that is followed, both of which are detailed in Section 4. The two chosen computing platforms are introduced in Section 5 and Section 6, where specific requirements and constraints are detailed, followed by a complete description of the design solution that is adopted. The results of such implementations are compared and analyzed with simulated and experimental data in Section 7. Finally, the paper draws to a close with some concluding remarks and directions for future work in Section 8.

## 2. State of the Art in Embedded Systems for Multi-Sensor Perception

On-road object detection, classification, and tracking has been a topic of intense interest over recent years [5]. The complexity of such tasks where vehicles and Vulnerable Road Users (VRU) (often pedestrians, motorcyclists, and cyclists ...) coexist in uncertain and heterogeneous environments makes the use of multi-sensor architectures very necessary for automated driving systems. Indeed, a variety of sensing modalities have become available for on-road vehicle detection, including radar, LiDAR, ultrasounds and computer vision.

While LiDARs have high accuracy under optimal conditions, wide angle coverage, and precise target location, their performance is less accurate in inclement weather and/or when dirt collects on a sensor lens [6]. Most radars use some form of beam scanning to determine whether targets are in the same or adjacent roadways, or in oncoming lanes. Microwave radars have a lengthy detection range and are able to operate in inclement weather, but they have a narrow field of view and are not robust enough for multi-target precise motion estimation [7], particularly around road curves. The images from a video camera can provide information on the presence of obstacles at short/medium distances [8]. These sensors have a wider field of vision and can recognize and categorize objects. However, they are not as reliable as radar when ascertaining depth-perception information. In addition, as humans vary significantly in size and shape, VRU detection is not robust enough, especially in crowded areas. Moreover, extreme lighting conditions (day/night) can dramatically reduce the effectiveness of the detection algorithms.

Modern ADAS applications use sensor fusion to take full advantage of the information that each sensor collects, so that the strengths of all these technologies can be intelligently combined. In some approaches, a radar or LiDAR is used to detect potential candidates, and then, during a second stage, Computer Vision is applied to analyze the objects that are detected. Other strategies claim the use of multiple-range sensors at a very low level to facilitate data fusion and ulterior object tracking. The Bayesian Occupancy Filter presents very good behavior under the latter paradigm. However, multiple-range sensors and other related grid-based approaches have typically been intractable for automotive embedded systems. Indeed, the commonly used MCU in the vehicle has insufficient processing power to process the various sensor inputs from multiple radars, cameras, laser scanners, and ultra-sonic sensors.

Future embedded solutions for perception will need to process high levels of throughput from heterogeneous sensors, providing real-time processing capabilities for computationally demanding algorithms while guaranteeing several non-functional properties such as reliability, real-time performance, low-cost, spatial constraints, low-power constraints, flexibility and short time-to-market [4].

The existing hardware (HW) technologies to cope with all the above requirements are as follows: (i) Application-Specific Integrated Circuits (ASIC), customized ciruits for particular uses, (ii) FPGA, (iii) GPGPU, (iv) Digital Signal Processors (DSP), and (v) microprocessors (μP). Table 1 summarizes the main advantages and drawbacks of each technology and provides some specific examples of ADAS applications where the perception of the environment plays a key role.

Note that most of these recent works use (mono or stereo) computer vision. The use of multi-sensor architectures and high-level functionalities is still very limited in the state-of-the-art embedded systems for intelligent vehicles. A limitation that is mainly due to both a lack of computing resources and very specific, rigid designs, unable to combine different functional components on a single computing platform. These difficulties have forced a shift from homogeneous machines relying on a single kind of fast-processing element to heterogeneous architectures combining different kinds of processors (such as MCUs, GPGPUs, DSPs, and FPGA), each specialized for certain tasks and programmed in a highly parallel fashion. Their weak points are poor optimization of available resources for performance and low energy consumption. Some examples of this new trend combine (i) multi-cores with FPGA for lane departure warning [20] and traffic sign recognition [21,22]; (ii) multi-cores with GPGPU [13]; and even, (iii) FPGA and GPGPU [23].

These new platforms allow us to consider traditionally prohibitive high-level processing for object detection and tracking using heterogeneous sensors. BOF real-time implementations are now realistic in this new context. One specific feature is that cells are independent, permitting dedicated hardware implementations leading to increased performance. Indeed, the cell independence hypothesis and sensor measurements tolerate loops in the Occupancy Grid algorithm that are implemented in a parallel manner. As a result, some prior works have explored the power of GPGPUs to implement different perception strategies based on occupancy grids, using multi-layer LiDARs [24] and fusing LiDAR and radar [25].

More recently, the integration of the occupancy grid multi-sensor fusion algorithm into low-power multi-core architectures has also been investigated [26,27]. These attempts use floating-point representation for probability estimation and fusion, but highly constrained embedded platforms will not often feature a floating-point unit. A fixed point design of an occupancy grid representation—BOF preprocessing step—was introduced by [28] to overcome that limitation. It shows good behavior, thereby opening the door to more HW-oriented implementations of the BOF (e.g., using FPGA). Indeed, in contrast to GPGPU architecture, FPGAs are designed for extreme customization. The current FPGA fine-grain architecture still takes advantage of irregular parallelism and non-standard data types. In addition, recent improvements are extending their applicability to different high-performance applications. This brings FPGAs closer in raw performance to state-of-the-art GPGPU-based SoC devices, as will be shown in Section 7.

## 3. Bayesian Occupancy Filter

Perception is one of the main processes that an autonomous vehicle has to perform. Within this process, data from the on-board sensors, typically multi-layer LiDARs and stereo cameras, are fed into the system for processing. The main purpose of the BOF framework when using those data feeds is to identify dynamic objects in a driving situation, estimating the position, trajectory, and velocity of each mobile object. These are then used for subsequent risk assessment and collision avoidance.

The BOF implementation is based on the work of INRIA (Institut National de Recherche en Informatique et en Automatique). A detailed description of its complexity and extension is unfortunately outside the scope of this paper. This section presents an overview of the framework; further details can be found in [29,30,31,32], also cited throughout the section.

**Figure 1:**
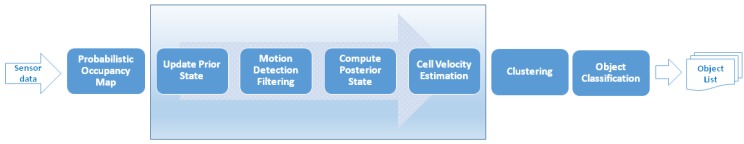
Bayesian Occupancy Filter process flow diagram and core system.

The process follows a series of sequential steps, where the output of each one is fed as input into the following one; see Figure 1. The emphasis on acceleration and parallelizable opportunities has been focused on the system core, which comprises the heaviest computational processes. This core updates the prior state, according to the dynamics of the vehicle, by applying motion detection filtering [29], by computing the posterior state from new observations, and by updating and computing new velocity estimations of the cells, described below in further detail. Other processes, such as the creation of a representation of the environment [31]—the probability occupancy map, the clustering process, to move from cell level to object level [32] and the identification/classification of each object [30] are not included in the initial acceleration and parallelization efforts.

**Figure 2:**
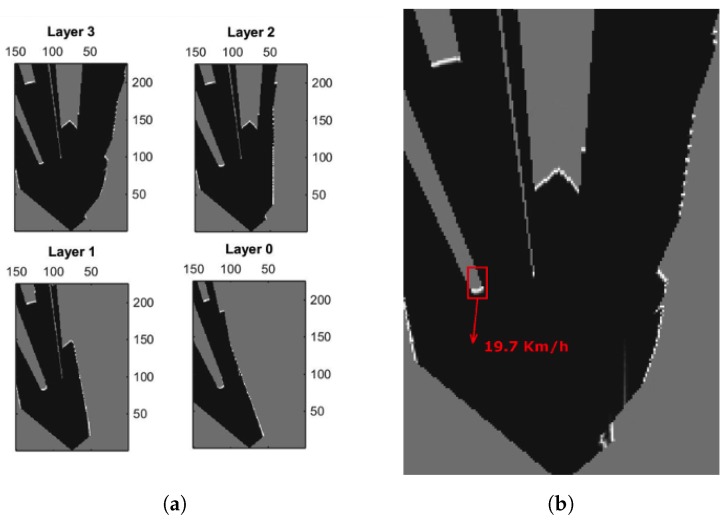
(**a**) Probabilistic occupancy map for each LiDAR layer, (**b**) integrated occupancy map. Dark areas represent empty space, white areas represent objects/occupied parts and unknown regions are in gray.

Initially, a representation of the environment must be created. A probabilistic occupancy map is built from sensor observations. The space in this grid-based map is discretized into M×N cells. Each cell contains the probability for the area that will be occupied. In the case of having multiple sensors, or a multi-layer LiDAR, an independent occupancy map is computed for each sensor/layer, see Figure 2. These multiple observations, and their corresponding occupancy maps, are then fused together into a unique representation of the environment, using a sophisticated weighted sum of observations, a mechanism known as Linear Opinion Pools [31]. The system uses that mechanism to compute an integrated probability value over the occupancy of each cell of the grid, given the opinion of a number of sensors. Also, a measure of the confidence for such estimations is also computed and taken into account to calculate the integrated occupancy probability for each cell. Factors such as confidence in each LiDAR measurement in frontal and rear areas of each potential hit and the inclination angle of each LiDAR plane layer—with respect to the road—influence the overall confidence level of each observation.

**Figure 3:**
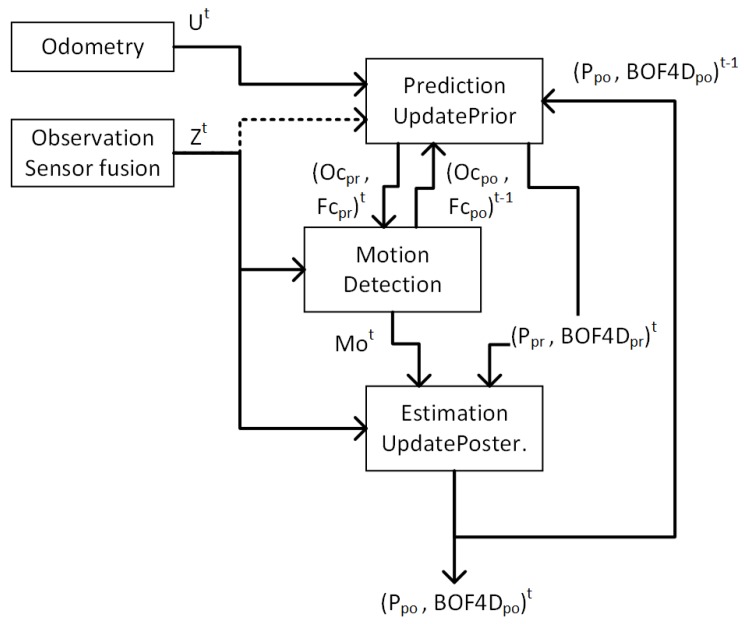
Bayesian Occupancy Framework general dataflow.

Figure 3 shows the BOF general dataflow. The algorithm calculates, for every time step (t), the state of the M×N cells in the occupancy grid (i.e., their Probability of occupancy P, and their distribution of velocity BOF4D), based on the previous state (t−1), the observed occupancy probabilities (Z), and the linear and angular velocities of the vehicle (U). The computation cycle takes place in two steps: UpdatePrior step and UpdatePosterior step. The results are notated with the corresponding subscripts ${}_{pr}$ and ${}_{po}$.

**UpdatePrior:** During the UpdatePrior step, a new prediction is created from the estimation of the previous state (P${}_{po}$, BOF4D${}_{po}$)${}^{t-1}$. This prediction is computed by transforming previous data according to the odometry, linear and angular velocities (U), over a given period of time. In a first process named ***CellTransform***, every cell, represented by the coordinates of its central point, is transformed according to this dynamic data and projected onto a new position on the predicted map.

Localization errors may occur, due to uncertainty, sensor inaccuracy and external hazards such as skidding, where the dynamics of the vehicle might not be precisely represented through proprioceptive information. A second process, ***OdomFix***, is applied to deal with that uncertainty problem and to update the Prior step data with greater accuracy. Instead of simply computing one transformation according to the vehicle dynamics based on sensor information, a series of potential transformations were calculated. Each transformation is then compared with the new observed occupancy grid (Z) and evaluated according to a score function. This function maintains a series of counters on the number of times each cell was observed as either empty (free) or occupied in the past (Fc, Oc) [29]. The candidate transformation with the highest score is then chosen as the prediction of the prior state information (P${}_{pr}$, BOF4D${}_{pr}$)${}^{t}$, and the free and occupied counters are updated according to this transformation (Oc${}_{pr}$,Fc${}_{pr}$)${}^{t}$.

**UpdatePosterior:** Once a prediction from the previous state has been computed, an estimation on the current state can be calculated, fusing that prediction with new observations. It is important to note this framework is designed to detect moving objects. However, most of the map or most of its cells turn out to contain information regarding static areas/objects. There is therefore no need to estimate the velocity for all those cells, which means that there is no need to update their velocity distribution, resulting in a huge saving of computation time. Therefore, a ***Motion Detection*** filtering step is all that is needed to make those savings through an heuristic function that classifies the cells as either static or dynamic, considering the values on either the free or the occupied counters. Therefore, if a given cell has been recorded as empty over some time in the past, in accordance with the free and occupied counter, and then appears as occupied in the most recent observation, it will be considered as a dynamic cell. A specific ratio has to be met between the number of times the cell was observed to be either empty or occupied, according to the counters, before it may be considered as dynamic. As a result, only those cells, marked in the Mo${}^{t}$ grid, will be taken into account to compute the posterior state (P${}_{po}$, BOF4D${}_{po}$)${}^{t}$.

**Figure 4:**
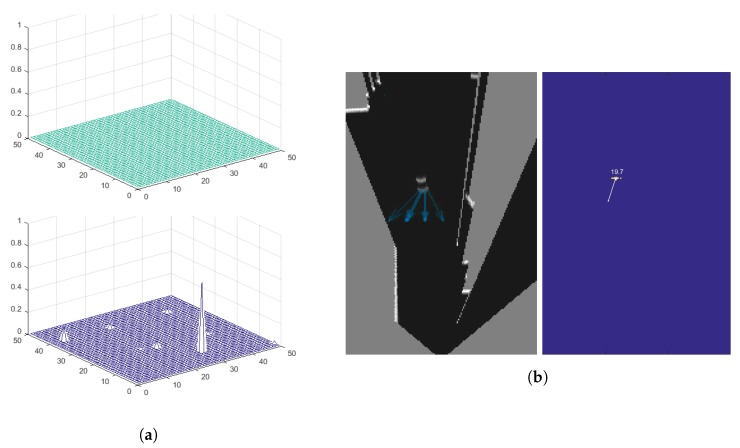
(**a**) Initial uniform probability distribution on the velocities (top) and convergence of probabilities (bottom), (**b**) clustering process grouping independent dynamic cells into objects.

A series of hypotheses on the velocity of every dynamic cell are proposed, to estimate the velocity and trajectory of dynamic objects from occupancy probabilities. An initial 2D uniform probability distribution on the velocity hypotheses, *vx* and *vy*, is created for each cell Figure 4a (top). The distribution is discretized into V×V values and initialized to 1/<number of cells> (1/2500 in the example). At each time step, besides updating the previous occupancy map to create a prediction, as previously described, a new probabilistic occupancy map is computed from new observations. They are then both fused together, obtaining an updated probabilistic occupancy map. The new observation data is also used to update the probability distribution on the velocity of each dynamic cell, propagating the probabilities using a ***Bayesian*** filtering mechanism [2]. In this propagation, each velocity hypothesis is updated according to the occupancy probability and probability distribution of the velocities of the *anterior*, which is the cell providing the prediction. The information on this cell is taken from the previous time step to establish whether a given velocity hypothesis is true for a given cell.

Over time, the distribution probabilities converge and, in some cases, one of them is prominent, see Figure 4a (bottom). The most prominent probability is then taken as the estimated velocity for that cell, provided its value is over a given minimum threshold to be considered, obtaining its module and trajectory from *vx* and *vy*. It is important to note that only the probability distribution of the dynamic cells are updated and propagated at each time step. The velocity hypotheses of the cells that are considered static cells are set to a uniform distribution.

So far the core of the BOF framework has been described. However, two processes still remain that are worth mentioning. The environment representation and tracking process have up until now been performed at the cell level. A process that is convenient, as it eliminates the data association and occlusion problems. However, the expected output of the system is a list of dynamic objects along with their associated position, trajectory, and velocity. Therefore, an object-level representation is necessary. To do so, a clustering process is applied to group independent cells into objects, see Figure 4b. A set of cells that belong to the same object can only be considered if they meet two conditions: the condition of spatial constraint, meaning that the cells have to be close enough to be considered part of the same object, according to a configurable distance parameter; and, similarity of their trajectories and velocities, implying that all the cells follow a similar trajectory at a similar velocity. Such a similarity is computed according to some configurable parameter relating to the maximum permitted differences in their trajectory orientations and velocity modules. The combination of both constraints avoids grouping together cells that belong to different objects, despite their physical proximity, as in the case of observing two cars at an intersection that will cross the path of the host vehicle from two opposing perpendicular directions.

Finally, accurate classification of a moving object in urban traffic scenarios is a key element for safe decision-making in intelligent vehicles. An object identification process, using a classifier combining grid-based footprints and speed estimations, aims to recognize each detected object at the scene and to classify them into different categories, such as vehicles or pedestrians [30]. As a result, the BOF framework delivers a list of dynamic objects that have been identified at the scene, along with their position and trajectory in relation to our vehicle, velocity and type of object.

All this information is then fed into a risk assessment module, part of the control subsystem, in charge of decision processes on directions and the computation of vehicle maneuvering.

## 4. Problem Description

The work described in the following 3 sections aims to compare different performance and functional metrics of the BOF design and implementation using on the one hand a Multi-processor System on Chip (MPSoC) with a GPGPU and on the other hand a MPSoC with an FPGA. This comparison will be conducted using synthetic data from a realistic simulation environment and experimental data from an automated vehicle driving on open roads.

The inputs to both designs are a set of measurements from the CAN (Controller Area Network) bus of the vehicle , including the linear velocity and heading of the vehicle in the given period of time (so-called odometry in Section 3), and those transmitted by the multi-layer LiDAR sensing the environment. The precision and the frequency at which these sensors update are specified in the following two sub-sections, where simulation and experimentation settings are described. In addition, the algorithmic output is expected to be the same in both approximations, namely a matrix of 240×160 cells including occupancy probability and 2D velocity estimation—from a discretized distribution of 15×15 values).

**Simulation:** SCANeR studio is a complete software tool meeting all the challenges of driving simulation. It includes models of sensors, vehicles and traffic interactions. With regard to the sensor models, the framework can simulate GPS, Camera, Radar, LiDAR and ultrasonic sensors. In particular for the LiDAR sensor, the simulation tool uses the ray tracing method to compute the results. Each ray is drawn in the graphic display and the rays are regularly drawn inside the detection arc (defined by the sensor position and a depth measurement).

**Figure 5:**
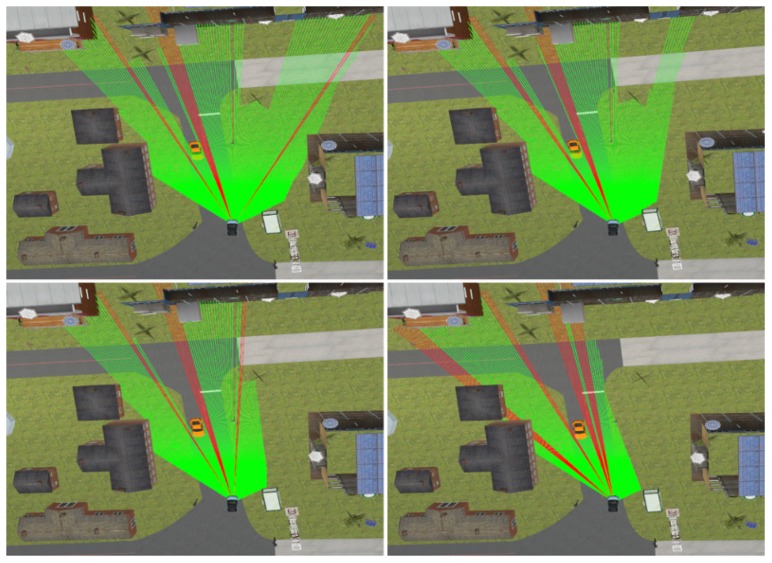
Fields of view of each LiDAR’s layer in each subfigure. Red rays are those that do not find any target, whose length is the LiDAR maximum depth (200 m for the IBEO Lux); green rays refer to those that collide in the 3D space, with a length that is the distance between the sensor and the target.

**Figure 6:**
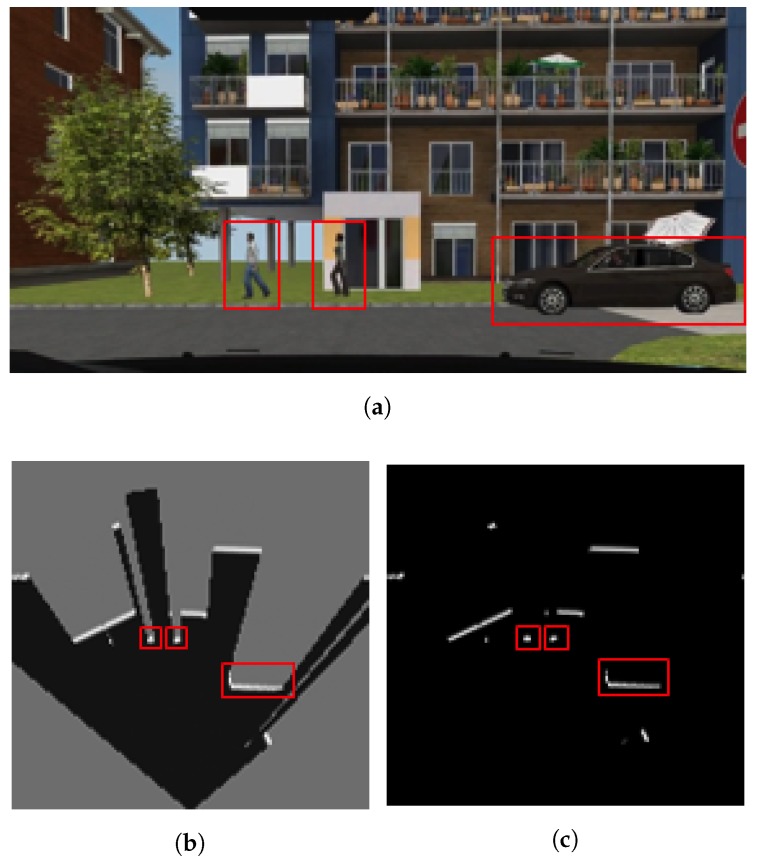
(**a**) Snapshot of a scene taken from the dataset where pedestrians and vehicles are detected (red boxes); (**b**) Image from camera, occupancy grid combining the 4 layers of the LiDAR; (**c**) the resulting set of footprints at the scene.

Figure 5 shows a simulated driving situation and how a vehicle, equipped with an emulated multi-layer IBEO Lux LiDAR, perceives the environment and other vehicles. This device consist of 4 different layers vertically separated at intervals of 0.8${}^{\circ }$ from each other, pointing from 1.2${}^{\circ }$ above the horizontal plane (top left image in Figure 5) to −1.2${}^{\circ }$ below (bottom right image in Figure 5). The data generated by this multi-layer LiDAR model is used to generate an integrated occupancy grid, as illustrated in Figure 2, and to extract the relevant footprints of the scene (Figure 6).

The dataset generated with this simulation framework is recorded from a pre-defined route of 650 m, which is completed at a variable speed after 120 s. The ego-vehicle always follows traffic regulations and it encounters other vehicles (7141 samples) or pedestrians (1861 samples). Note that all of the 1554 observations in the datatset may contain one or several of these samples. The LiDAR frequency is 12.5 Hz, while the longitudinal and angular speed of the vehicle is measured at 25 Hz.

**Figure 7:**
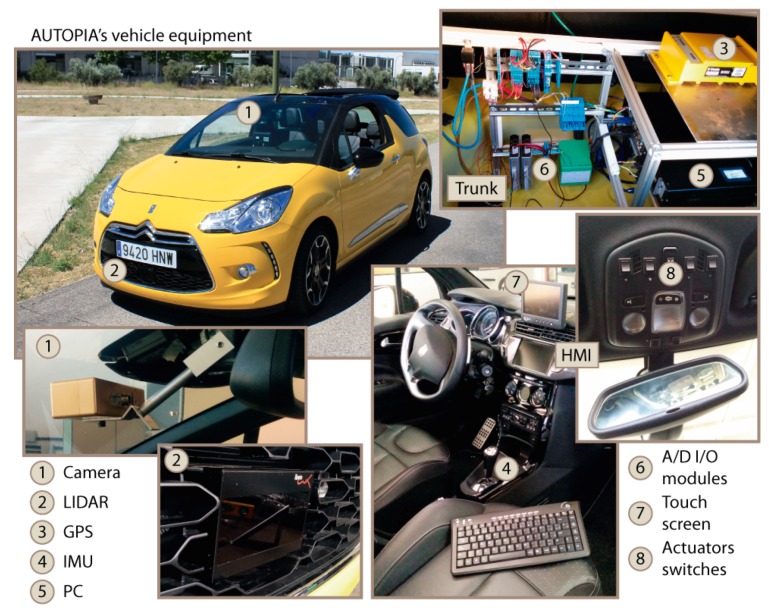
Automated vehicle architecture.

**Experimentation:** A dataset has been recorded using an automated vehicle, to complement the results obtained using the simulation environment. The picture on the left-hand-side of Figure 7 shows the architecture of the vehicle, where the most relevant equipments are zoomed and numbered (note the IBEO Lux LiDAR in number 2). In addition to these visible parts, the SW architecture integrates the data from internal sensors (steering wheel, yaw rate, wheel speed, lateral and longitudinal accelerometers) that circulate through the CAN bus. The sampling frequency of these data is identical to those described in the simulation framework, parameterized to be consistent with the real on-board sensors.

**Figure 8:**
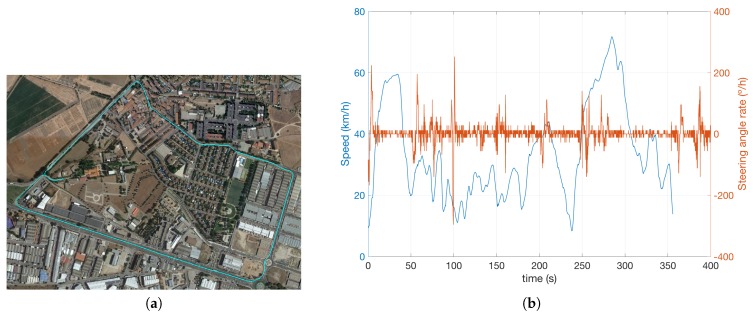
(**a**) Path followed by the vehicle (**b**) Speed and steering angle of the vehicle.

The experimental recording consisted of a 3350.3 m urban and inter-urban route (Figure 8a), during 356 s, where different types of vehicles and pedestrians appear in the vehicle driving scene throughout the 4447 cycles/observations. As can be appreciated in Figure 8b, the vehicle speed never exceeds 70.8 km/h, but there are several stretches where there is a meaningful steering angle variation (a maximum of 284${}^{\circ }$/s), leading to demanding angular speed for the BOF computation.

**Validation:** A common methodology was used to validate the results of the designs described in the following 2 sections. First, an occupancy map is computed from LiDAR hits combining the different layers (see the first functional block in Figure 1). Then, a reference or gold code is implemented in Matlab that includes the different functional blocks contained within the box in the same process diagram. The output of this code, generated by Matlab R2015b (Mathworks, Natick, MA, USA) with double precision, serves as groundtruth for the BOF outputs in each design (both for FPGA and for GPGPU).

Given this methodological framework, each technology needs different tools to estimate the computing and memory resources needed at each implementation phase. In the case of the GPGPU, nsight was used to develop the functionalities and to test them, while profiling was conducted with the NVIDIA Visual Profiler. As regards FPGA, Xilinx Vivado 2016.3 (Xilinx, San Jose, CA, USA) tools were employed for iterative design, validation, profiling and synthesis.

## 5. Design on GPGPU

The platform used for this implementation is the Nvidia Jetson TK1. It includes an MPSoC Tegra K1 (TK1) with a quad-core processor, a GPGPU with 192 CUDA cores and 2 GB of DDR3L RAM. The processor consists of 4 Cortex A15 cores, clocked at 2.32 GHz. The Kepler GPGPU contains 1 streaming multiprocessor (SMX), at a maximum frequency of 835 MHz. The SMX is the architectural block around which GPGPU scalability is built. Each SMX has its own cores, its shared memory and L1 cache, register file, load/store units, special functions units and its warp schedulers [33]. Warps are groups of 32 threads. Each SMX receives thread blocks from the kernels, the functions of which will be executed in the GPGPU. When a kernel is launched, the size of the grid and the size of each block must be defined. These blocks are distributed among SMX and their instructions are scheduled in the thread scheduler, which selects one from among the available warps to execute each instruction. Every thread in a warp executes the same instruction, if not disabled because of a branch divergence. Several clock cycles take place from the time a given instruction starts its execution in a thread until it ends. This latency depends on the GPGPU architecture and is 11 clock cycles on this platform.

Among other components, each GPGPU core is formed by an integer and a floating-point unit. Both have a 32-bit word size. However, the performance of the floating point arithmetic unit is better than the integer arithmetic, see Table 2. Also, the GPGPU of this platform has a lower power consumption of approximately 10 Watts, despite its similarities with desktop GPGPUs. A main difference between the SoC architecture and discrete GPGPUs is that the principal memory is shared between processor and GPGPU in SoCs.

### 5.1. Data Structures and Memory Hierarchy

As indicated before, data is stored as either floats or integers. A float is the most widely used for arithmetic computation, because of its higher throughput. Integers are mainly used for matrix indexing, avoiding type conversion from float to integer. The loss of precision from the original FP64 (double precision floating point format) code to the CUDA FP32 (single precision floating point format) version lead to some minor differences in the computation of probabilities, with little influence on the final results.

Modern GPGPUs are designed with a high variety of memories, each one with its own peculiarities, see Table 3. This diversity allows the developer to place each type of data in the most appropriate memory to achieve the highest possible throughput. These memories can be on-chip, with higher bandwidth and lower latencies, or off-chip, allowing higher amounts of memory. Also, each type of memory has its own scope and persistence, varying from application to thread lifetime. Given the size of our maps and the requirement of persistence, they have to be stored in off-chip memory. Our maps and table sizes are less than 7 MB. Constant memory is not an option because it can not be modified from the GPGPU. Both texture/surface memory and global memory are cached. However, the low number of texture mapping units per SMX and the complex process of data readings means that global memory achieves a better performance than texture/surface memory. By default, the L1 cache for global memory is disabled, and it is necessary to activate the NVCC argument when compiling the program, significantly improving the performance of some kernels.

In our program, several Look Up Tables (LUTs) are used. Some of them are declared in Constant memory and others in global memory. The reason behind that arrangement is the way they are read, as the access patterns of both memories differ. As threads in the same block can read from the shared memory space reserved for that block, it is very suitable for thread interaction. Depending on the kernel needs, both the L1 cache and the shared memory space can be resized. The total amount of memory is 64 kB. By default, the size of both memories is 32 kB (32-32). When more shared memory is necessary, it can be configured as 16-48. Likewise, when the kernel needs more cache, it can be configured as 48-16. Shared memory is organized in 32 memory banks. The best performance is achieved when each thread in a warp accesses different banks. In case more than one thread has to access the same memory bank, a conflict arises and requests are serialized.

### 5.2. Optimizations

The main goal when optimizing an algorithm is to achieve the highest possible throughput. It can be reached following a sequence of multiple parameter optimizations. One of the first optimizations has to do with the kernel design. Each kernel is composed of a grid of blocks and each block contains multiple threads. Our goal is to perform the computation that has to do with each cell in the map in an independent thread. It can be easily achieved in most kernels given the independency of the cells. When there is dependency between cells, synchronization between threads must be implemented, penalizing the throughput.

The dimension of the kernel is also a parameter that affects the throughput. As each SMX can only manage a certain number of blocks and threads, with small blocks, the SMX will limit the number of blocks, and therefore the number of threads which are under execution at the same time. It implies that the occupancy, meaning the percentage of active warps, decreases.

Following the goal to achieve the highest throughput, it is preferable if every core executes an instruction every cycle. However, memory latencies mean that every core cannot always do so. Strategies to increment the Instruction Level Parallelism (ILP) can reduce the number of those non-productive cycles: a given thread can therefore perform computations related to more than one cell. In that way, if the warp scheduler has to wait because the previous instruction has yet to end, an instruction of the same thread but for a different cell can be scheduled, as cells are independent. The main drawback of this strategy is that computing more than one cell in a given thread increases the amount of necessary resources, and in case it exceeds the previously mentioned limit, the occupancy will decrease. However, a lower occupancy will not always mean a lower throughput. Therefore, multiple configurations have to be tested when designing the kernel to find the optimal one.

A general strategy to increase the throughput is to divide each kernel into simpler and lighter ones. As the number of dependent instructions decrease, it is possible to obtain lower latencies. However, that may not sometimes work as expected, as caches play an important role in this strategy.

**Figure 9:**
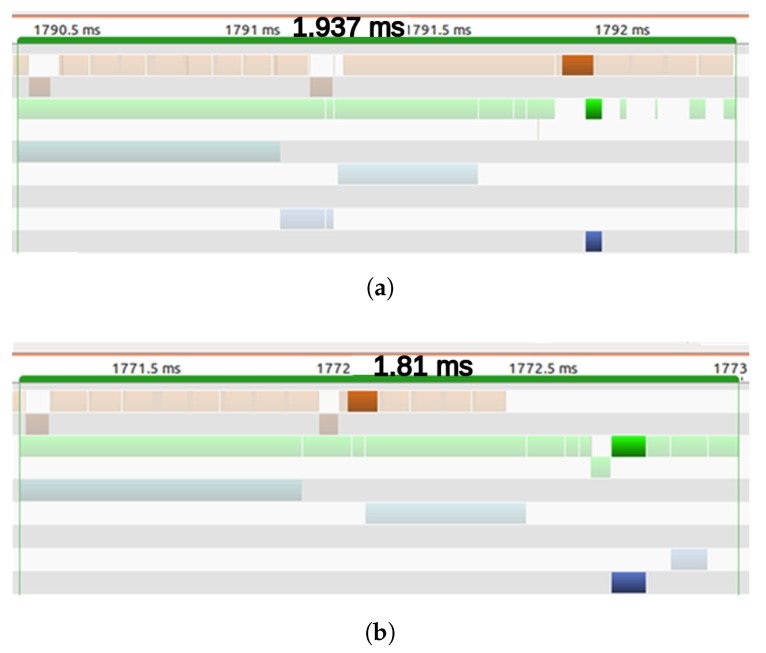
Launch time (orange) and execution time (green) for both strategies: (**a**) performing Motion Detection Filter first and (**b**) launching the maximum number of threads.

As indicated in the previous section, rather than compute the Posterior state for every cell, a Motion Detection Filter classifies cells into static and dynamic, so the Posterior state will only be computed for the dynamic cells. At this point, two different strategies were developed to launch the kernels: (1) Performing the Motion Detection Filter on the whole grid (map) and then launching the Posterior State kernel with the dimension in accordance with the number of dynamic cells; or, (2) launching the maximum number of threads and letting each one decide if the task should be performed, based on the previously calculated number of dynamic cells. For the latter strategy, the next question may arise: if each thread decides whether to compute the Posterior state, why is it necessary to launch all of the threads? In addressing this question, it is important to note the differences between these two concepts: launch and execution of a kernel. The kernel launch is a preparation step. First, block and grid dimensions are defined; blocks are then generated with these parameters and distributed between the SMX. The execution of the kernel happens when the actual instructions are executed in the GPGPU. Now, returning to the first strategy proposed, where just the exact number of blocks are launched, the processor has to wait until the Motion Detection Filter has finished launching the kernel, and only when it has been launched can the execution start, see Figure 9a. For the second strategy, as the parameters of the kernels do not change over time, the launch can be done at any time. As the kernel does not start its execution until the Motion Detection Filter has finished, see Figure 9b, all threads have access to the result of the Filter when the execution starts. With this strategy, the execution can start immediately after the filter has finished while the first strategy has to wait until the launch step is done.

Having run both strategies 500 times, it was shown that the first strategy runs faster than the second one for a low number of dynamic cells. However, as the number of dynamic cells increased, the launching time in the first strategy penalized the performance and slowed it down. As the number of dynamic cells was relatively low in all our experiments, the first strategy was adopted.

## 6. FPGA Design

Contrary to the GPGPU design flow, based on a predefined hardware-software architecture, FPGAs are fully customizable and the FPGA architecture can be adjusted to the algorithm requirements. To do so, they count with three types of resources: logic cells (small Look Up Table memories and Flip Flops), block memory (blocks of static RAM), and arithmetic cores (DSP slices). FPGA design requires highly skilled engineers with a profound knowledge of the underlying hardware that is needed to build an optimized architecture to fit the algorithm in the available resources. Fortunately, Xilinx offers a software-friendly design flow based on a C-to-hardware compiler called VivadoHLS [34]. This compiler support synthesis directives and pragmas to automatically transform C/C++ code to hardware blocks that are described at register-transfer level, also known as RTL cores. The synthesis means that low-level details of logic and interfaces to memories and buses are transparent to the programmer. Architectural design decisions can be changed just by adding or removing those directives. However, code re-factorization was necessary to exploit the inherent parallelism of the BOF.

The platform used for FPGA implementation was the Enclustra ZX1 [35]. This board is based on Xilinx Zynq XC7Z035 SoC device, which is equipped with dual-core ARM Cortex-A9 processor integrated with 28 nm Kintex-7-based programmable logic. The processor includes two levels of cache, functions up to 1 GHz, and is tightly coupled to the FPGA fabric by means of 4 high performance ports. Dual-core processor and FPGA fabric share a 32-bit external memory interface connected to 1 GByte of DDR3L SDRAM. The programmable side of the device is populated with 275 K logic cells, 900 DSP slices and 500 blocks of RAM (36 kB each). Every DSP slice implements one fixed point multiplier (18×25 bits) and one 48-bit adder which can be configured for Multiply&Accumulate operations.

### 6.1. Data Structures and Memory Hierarchy

The BOF algorithm works through a significant number of large data structures (2D and 4D matrices). The FPGA platform uses different memory technologies to store all these data. On the one hand, external DDR memory offers a very high bandwidth when large blocks of contiguous data are accessed. However, the number of wait cycles is unpredictable for random accesses and degrades the final performance. On the other hand, built-in block RAM offers less capacity, but can be configured (partitioned and reshaped) to feed data at the pace of the internal pipelines avoiding stalls in the dataflow. Moreover, they offer real dual ports, which allow concurrent access from different hardware blocks to the same data structure. Consequently, if the throughput of FPGA designs is to be improved, the retention of data in on-chip bram caches for as long as possible is crucial.

**Figure 10:**
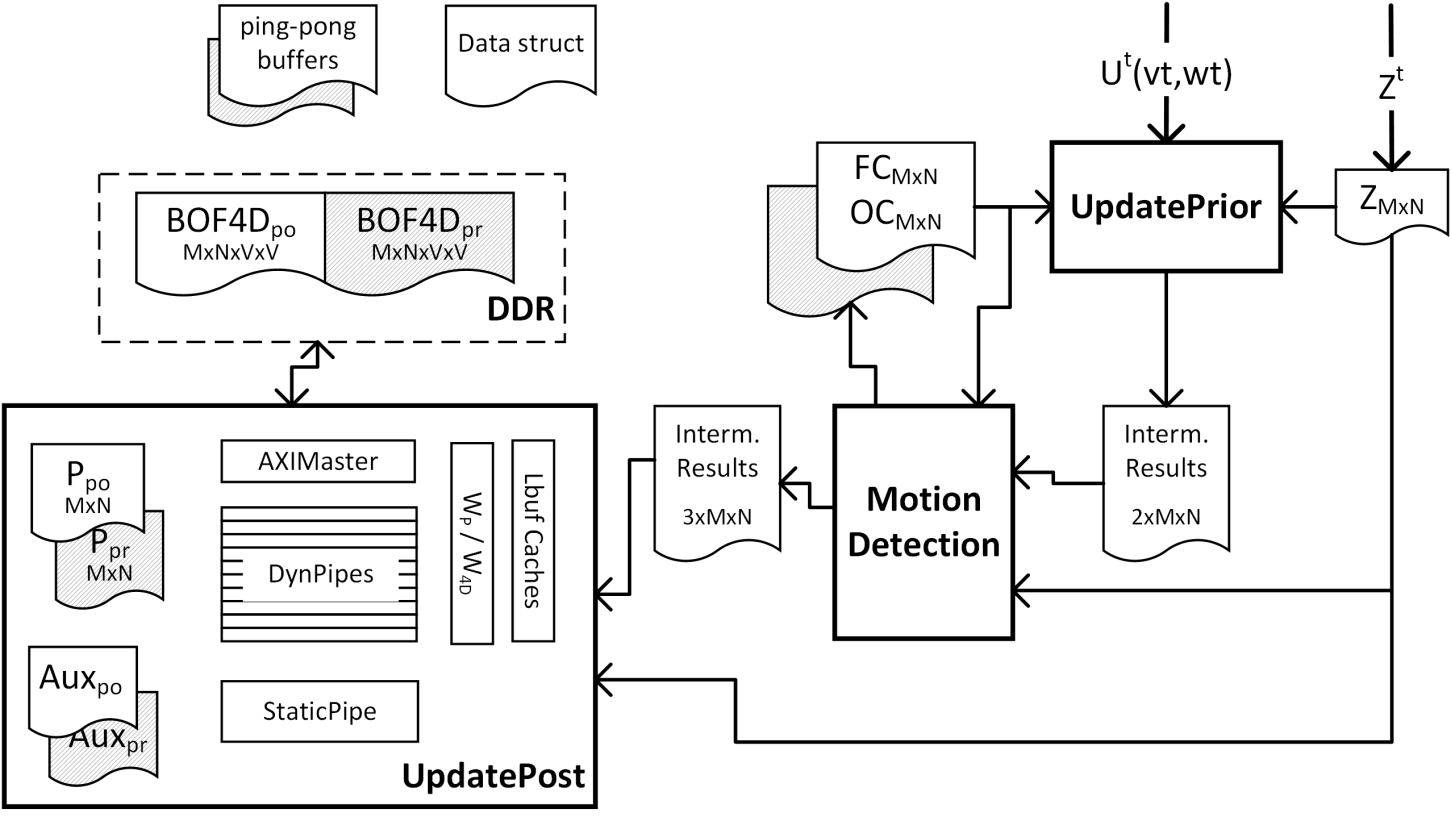
Main blocks of the FPGA design.

Following this principle, Figure 10 shows a general view of the customized architecture proposed for accelerating the BOF algorithm. It comprises three hardware blocks corresponding to the main BOF processes. During a computation cycle, t, the blocks work sequentially on different data generating intermediate results to be consumed by subsequent blocks. Those data are shared between blocks by means of IntermResults structures. Additionally, other information has to be preserved between consecutive computation cycles, which is the case of the cell state (P${}_{po}$, BOF4D${}_{po}$), Fc/Oc counters, and auxiliary data for tracking static cells and new cells entering the grid. The posterior information produced in the previous cycle (t−1) becomes the prior information for the next cycle (*t*). The ’ping-pong’ buffers used for storing these sorts of data are composed of two identical structures, which enable simultaneous read (from the ping buffer) and write (to the pong buffer). In this way, memory contention is avoided and throughput is significantly improved. At the end of the computation, rules are exchanged between ping and pong preparing the architecture for the next cycle.

In addition to the huge number of data, ping-pong buffers duplicate the consumption of the scarce BRAM (FPGA built-in blocks of RAM memory) resources. A study was therefore carried out for optimizing the word length of the data prior to the implementation phase. For comparative purposes, the simulated dataset was processed by the reference code in FP32 representation, and the results of each iteration recorded. These results were compared, frame by frame, with those obtained in a bit-accurate simulation of the proposed architecture. In this version, internal operations were performed in floating point arithmetic, meanwhile P and BOF4D data were truncated to arbitrary sizes prior to their storage. Simulation showed that a truncation to 16 bit produced a deviation from reference results close to the quantization error, i.e., 2${}^{-15}$. So there was an average quadratic error of below $5\times 10^{-5}$ in the case of velocity probabilities and under $5\times 10^{-3}$ in the case of occupancy probabilities. Both errors can be considered suitable for real applications [28].

Moreover, Xilinx FPGAs lack floating point units, so a second study was undertaken considering the effect of fixed point arithmetic on internal operations. The study revealed two independent sources of error: the UpdatePrior process is responsible for errors associated with object locations, while the UpdatePosterior process yields errors in the calculation of occupancy probabilities and the distribution of velocities. Several simulations were performed with a fixed point version of the architecture and compared to the reference data. The results showed an accuracy, expressed as a percentage of correctly transformed cells, of 100% for 48-bit width and 98% for 36-bit width. Although the latter consumes less FPGA resources and could be considered in some implementations, the maximum precision format (48-bit) was selected in this work to allow a fair comparison with the GPGPU version. Furthermore, 48-bit representation also provided an acceptable dynamic range to the velocity distribution during the simulation.

As shown in Figure 10, after applying these optimizations to mid-range FPGAs, only BOF4D structures needed external DDR storage, because of the large capacity that is required (M×N matrices of V×V values).

### 6.2. Architecture Optimizations

Once the fixed point version of the BOF architecture was stable, several optimizations were applied for improving the performance of the implementation. Three design rules were followed to squeeze the FPGA potential: pipelining, data reuse, and core replication.

Due to the inherent parallelism of cell computation during the first two BOF processes, the corresponding blocks are internally pipelined to increase their throughput. Therefore, multiple cell coordinates are simultaneously calculated in the UpdatePrior block process by means of a pipelined CORDIC (COordinate Rotation DIgital Computer) core—iterative algorithm which approximate the result bit by bit using only shift and add operations.After its initial latency, the core produces a new coordinate transformation per clock cycle. Similar throughput was reached in the Motion Detection block after pipelining optimization. However, data dependencies between both processes prevent cascading of the pipelines, therefore the global latency of the blocks are the sum of their individual latencies.

In the case of UpdatePosterior block, asymmetric computation takes place depending on the type of cell to process. Therefore, two different pipelines were designed (see Figure 10). On the one hand, if a single dynamic cell is to be computed, it is necessary to read the state of the antecedent cells i.e., a window of V×V values of P${}_{pr}$, plus a window of V×V velocity rows (each row from a different BOF4D${}_{pr}$ velocity matrix). In the case of P${}_{pr}$, an array of V−1 lines are arranged in an special cache (Lbuff) to avoid repetitive accesses to antecedent cells shared by overlapped windows. This structure is updated cycle by cycle with the values sequentially read from the P${}_{pr}$ memory, feeding a V×V sliding window (W${}_{P}$). The sliding window is fully mapped in registers, providing the necessary values to process the P${}_{po}$ of any given cell in just one cycle. Similarly, the W${}_{4D}$ sliding window exposes every clock cycle, all necessary data for computing a whole column of the BOF4D${}_{po}$ matrix. In this case the operands, and the results are directly read (written) from (to) DDR memory by means of a bus Master (AXIMaster block). As a final optimization, the dynamic pipeline was duplicated *V* times to produce a whole row of results per clock cycle.

Table 4 shows the resource utilization of the proposed design. As can be seen, UpdatePosterior block is more resource intensive in all cases. In this core, the BRAMs are mainly consumed for the sliding window caches, meanwhile DSP and logic resources (LUT and Flip-flops) are dedicated to the implementation of 15 parallel dynamic pipelines of complex calculations. Both stages, i.e., UpdatePrior block and Motion detection, on the one hand, and UpdatePosterior block, on the other hand, were implemented and separately verified on a ZX1 platform, as the total resources exceed the maximum available in the XC7035 device. However, the implementation of the whole architecture is quite feasible by selecting another mid-range FPGA as the target device such as XC7045 or higher.

## 7. Comparison of GPGPU and FPGA Designs

Actual GPGPU and FPGA devices have different architectural resources. GPU are specialized in floating point arithmetic, and it is guaranteed that CUDA cores will work at maximum clock frequency independently of the workload. Meanwhile, FPGAs only provide fixed point units, the work frequency of which depends on the design. Even though they can be arranged for floating point operations, the correlation between CUDA core and DSP slices may not be defined, since their gate count is completely different. Furthermore, GPU cache hierarchy is fixed and optimized for multiprocessors, on the contrary FPGA built-in memories are not hierarchic and are fully configurable. Taking into account those difficulties to find quite equivalent parts, and trying to set comparable platforms as far as possible, two technologically similar devices were selected. In our case, both were presented by their companies as reference platforms for ADAS implementation, using equal process technology (28 nm) and very near release dates (Zynq FPGAs were officially released in the 2nd quarter of 2013, but mid-range devices were not available until the next year).

Additionally, a second circumstance was considered in our study. Both the design flow and the test procedures for FPGA differ from those for GPGPU, requiring unrelated development efforts to reach similar figures. On the one hand, GPGPU design follows a software development methodology, where the designer focuses on code re-factorization and refinement to improve performance. The optimization of memory transactions, one of the main bottlenecks in parallelization, is adjusted in the code by means of library functions and data reorganization. Any modification can be compiled in seconds and evaluated in real-time on the final platform. In addition, the process takes advantage of debugging facilities and software profiling tools, which improves the observability of the implementation without additional code modifications. FPGA design flow is a more complex and tedious process; although the use of High Level Synthesis tools, like VivadoHLS, accelerates functional verification, it will not guarantee the correctness of the final result. After the synthesis process, which may take minutes/hours, a second verification is needed to co-simulate the outcome RTL code against the software testbenches. Co-simulation offers cycle accurate estimations of the performance, but may take hours over a design with the complexity of BOF. As a matter of fact, the co-simulation of the UpdatePosterior block for processing just one frame took around 30 min, dependent on the number of dynamic cells. Moreover, exhaustive testing was required before the Place&Route phase prior to deployment in the final platform. Taking this into consideration, and in order to provide a fair comparison, an equal development effort of 6 moth-man was set for both designs. During this development effort, both versions of the BOF were developed and tested using the same datasets and reference code. The final implementations are summarized in Table 5. A direct comparison between the results is performed, in the following subsections, in terms of accuracy and performance.

### 7.1. Accuracy

The accuracy of each implementation was calculated as the mismatch between the results obtained with the reference Matlab code running the simulated dataset and the results offered by the corresponding GPGPU or FPGA version. BOF accuracy is influenced by two factors of a different nature. First, the error associated with coordinate transformation, which is produced by the UpdatePrior computation. Second, the error in the calculation of occupancy probabilities (P) and velocity distributions (BOF4D), which is produced in the UpdatePosterior step. Each error was analyzed individually in the following paragraphs.

**Figure 11:**
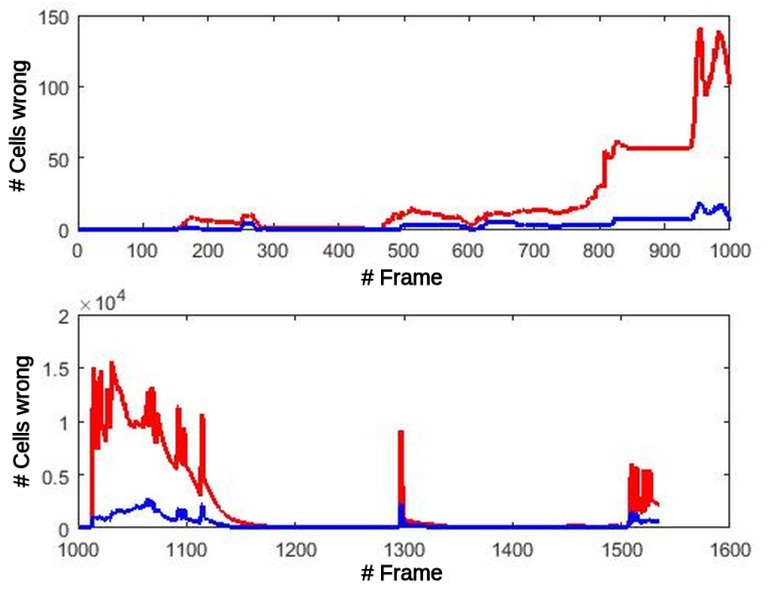
GPGPU errors after Prior stage. Red: counters of non-occupied cells (Fc). Blue: counters of occupied cells (Oc).

Coordinate transformations performed in the UpdatePrior block are prone to errors because of reduced precision but, fortunately, it is independent of the cell state and therefore immune to errors in the probabilities produced by previous iterations. In this computation step, the lower number of bits of each variable can be determinant in transforming a point to the right cell or to its neighbor when the transformation with full precision is very close to the border of these 2 cells. When an error occurs, it will exist until the cell exits the map. In Figure 11, the number of Oc and Fc cells is shown that have a different value in the GPGPU version (single precision floating point FP32) from the reference code (double precision floating point FP64), both processing the simulated dataset. This figure in no way means that all those errors were committed in each frame. In fact, the number of errors for each frame are divided into two groups: errors committed because of transformations for this frame, and cells with a wrong value that are still in the map from previous frames. Oc and Fc errors remain very low, under 150 erroneous cells (0.39% of the cells), until frame 1014. At this point, errors in counters modify the selection of the most suitable transformation in the OdomFix, producing an incorrect translation of one position for all the cells in the grid. From this frame, the differences increase dramatically, but it simply means a systematic positioning error of ±1 cell.

**Figure 12:**
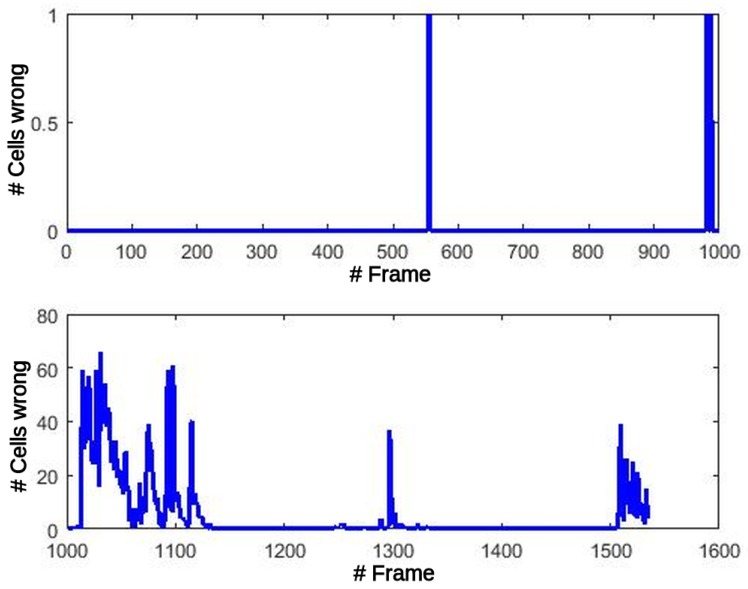
GPGPU errors after Motion detection stage.

The propagation of these errors to the Motion detection stage might produce misclassification of the cells between static and dynamic types. Figure 12 shows this effect at the output of the Motion detection block. The number of errors before the critical frame are less than 5 in total. After this frame, the number of errors increase and remain under 70 errors/frame, at close to zero most of the time.

Finally, analyzing the UpdatePosterior step, two sources of error can be appreciated. In the first place, the state of the antecedent cells, which could be incorrect, because of Mo misclassification and/or erroneous coordinate transformation. In second place, the intrinsic errors produced in the UpdatedPosterior block, due to the limited precision of the internal operations. The influence of each source on final inaccuracy is difficult to determine, but clearly both of them should be minimized to achieve the most reliable results. The relevance of the inaccuracies in the outcome of UpdatePosterior block was analyzed, for each cell in every frame of the data set, comparing the module and angle of velocity corresponding to the higher value in the probability distribution, i.e., the most probable speed and direction for the cell. Table 6 shows the mean and standard deviation of these errors. As can be seen, the effect of errors propagated from the previous stages remain, and increases the mean error from frame 1014.

Contrary to the GPGPU version, FPGA offers a perfect match in the case of Oc counters, and just one (1) error in Fc all over the whole dataset. Moreover, errors after the Motion detection stage are zero for all the frames. This could be found contradictory since FPGA calculations were approximated with CORDIC functions and fixed-point arithmetic. However, this fact reveals one the main advantages of this technology, since datawidth can be adjusted arbitrarily to fit the algorithm requirements (up to INT48 in this case), meanwhile data representation in GPGPU is predefined by fixed hardware to FP32 or INT32. Consequently, there are no errors propagated to the UpdatePosterior block and its final results are influenced only by the quantization of the internal operations. As can be seen in Table 7 the mean error in module and angle remains low and constant throughout the whole dataset.

### 7.2. Performance

The performance of both versions was calculated as the latency (in milliseconds) to process a frame. This metric is coherent with the usual integration of the BOF in ADAS systems. As depicted in Figure 1, BOF is an intermediate step among different processes, and all of them must be executed before the arrival of the next observation (Z, U). In our datasets, a new processing step starts every 80 ms (12.5 Hz), so BOF processing needs to be accelerated as much as possible to leave enough room for the other processes.

**Figure 13:**
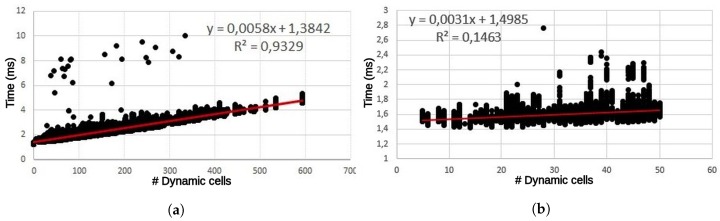
Latencies of GPGPU version vs. number of dynamic cells. (**a**) For all number of dynamic cells of the dataset; (**b**) Zoom from 1 to 50 dynamic cells.

**Figure 14:**
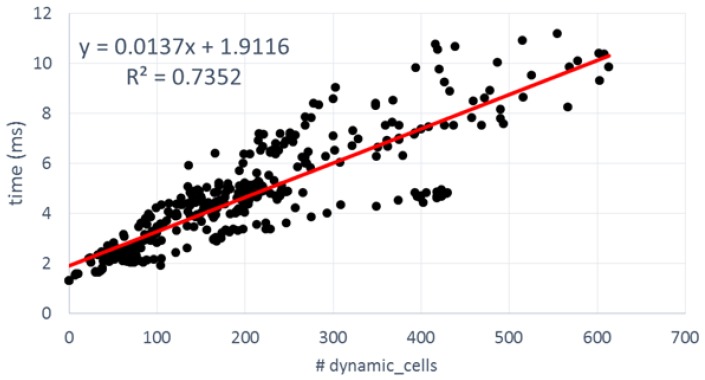
Latencies of FPGA version versus number of dynamic cells.

As explained in previous sections, the latency of BOF computation is the summation of the individual latencies of the three main processes. The first two latencies are directly related to the number of cells in the grid. Hence, they are constants along all the frames of the dataset. However, the third is variable since the number and complexity of the calculations, in UpdatedPosterior block, are quite different depending on the nature of the cell (static or dynamic). Taking into account this dependency, latency was measured for all frames in the experimental dataset and depicted in Figure 13 and Figure 14 as a function of the number of dynamic cells.

The latency per frame in the GPGPU version (Figure 13) shows a clear linear dependency with the number of dynamic cells. The fitted line can be divided into two regions: while the number of dynamic cells are below 50, the equation is shown in Figure 13 on the right. In contrast, when the number of dynamic cells are higher than 50, then the equation is the one shown in Figure 13 on the left. The differences between the slopes in both regions are originated by the launching times and execution times. In the first region, execution times are hidden by launching times, while launching times in the second region are hidden by execution times. In the case of 0 dynamic cells there is no launching or execution times and the latency is 1.34 ms. The majority of frames, around 99%, present fewer than 400 dynamic cells and a latency of around 4 ms. There are some outliers, approximately 0.00003% of the frames, which took more than 6 ms. This delay is produced by the operating system and can be explained by the context switches during the testbench execution. The distribution of latency between the main processing steps can be seen in Table 8.

Figure 14 shows the equivalent FPGA numbers. It is worth mentioning that Enclustra ZX1 board, which includes a low-cost XC7035 device, was used to measure the latencies in terms of the number of cycles. Then the results were extrapolated to a high-performance XC7045 device running at 175 MHz. The design was implemented with Xilinx Vivado 2016.3 for corroborating a maximum supported frequency of 176.4 MHz.

Several differences can be appreciated when compared to the GPGPU results. In first place, latencies are longer than in the GPGPU counterpart. From among 200 dynamic cells, most frames take more than 4 ms before they are processed. The linear fitting of the FPGA points has approximately twice the slope of the GPGPU, so roughly speaking we can say that GPGPU offers a 2× speedup over FPGA. In second place, the dispersion around the curve is greater than in the previous case: R-square is 0.7352 vs. 0.9329 in GPGPU. The software side of the FPGA SoC was implemented as a stand-alone, so there are no interferences with any operative system to generate this dispersion. Table 9 offers additional information to explain the differences. As can be appreciated, the latencies of the first two blocks present no dependencies with the types of cells under process. In this case, the execution times of UpdatePrior and Motion detection are quite invariant because, in addition to the low interference coming from the bare-metal software, all data structures are cached in an internal BRAM, which present a deterministic access time without variable latencies. Only the UpdatePrior step offers better performance in the FPGA version (0.4245 ms vs. 1.253 ms).

The design presents some bottlenecks mainly in the UpdatePosterior block that explain the loss of performance. Due to the limited development effort, access to external DDR memory was not optimized by means of separated read and write channels, neither was the possibility of increasing the width of the data bus (by up to 128 bits) explored. In addition, BOF data reuse is not intensive and uniform enough to take advantage of the sliding window caches. Only when dynamic cells are consecutive will the data cache be reused. In any other case, the sliding window must be fully refilled accessing the external DDR memory and significantly increasing the computing time. This fact explains the dispersion of the points. For instance, the processing time for a frame with 199 dynamic cells can take from 3.36 ms to 6.36 ms. When the number of dynamic cells increases near to 400, the time interval starts at 4.43 ms and rises to 9.82 ms.

## 8. Concluding Remarks

A comparison of designs for Bayesian Occupancy Filters in MPSoC systems has been presented in this paper. The analysis has considered 2 different technologies: FPGA and GPGPU, for which ad-hoc implementations have been performed. From an initial reference implementation in Matlab, effort, accuracy and performance have been compared using both simulated and experimental datasets.

The main conclusions of this in-depth analysis can be summarized in the three following aspects:

- FPGA technology permits a much more tailored design, able to adapt the word length to each specific data processing and data transfer process, thus obtaining unparalleled accuracy.
- The nature of the Bayesian Occupancy Filter appears better adapted for GPGPU architectures in terms of latencies, particularly in the Update Posterior phase, where the memory accesses for non-intensive data use penalizes the performance of FPGA.
- For a development effort of 6 months-man, GPGPU design offers an overall speedup of 2× compared to FPGA. Only UpdatePrior process is more efficient, close to 3×, in the FPGA design in terms of performance. Further improvements are possible for the UpdatePosterior process, which will nevertheless require lengthier development times than in the case of GPGPU.

Future work will be oriented towards applying the lessons learnt in this work to optimize the dataflow in upcoming computing platforms where GPGPU and FPGA are integrated on the same chip. In addition, stereo vision will be also fused with LiDAR to obtain a more reliable occupancy grid. To that end, a specific FPGA-based disparity map will be implemented, targeting an overall work cycle of 50 ms.

## Figures and Tables

**Table 1:** Examples of latest-generation embedded ADAS using different HW technologies.

Tech.	Pros	Cons	Examples
ASIC	High performance	Expensive for prototyping	Disparity maps [9]
	Low-power consumption	Not reconfigurable	Object and lane detection [10]
FPGA	Low-power consumption	Poor for serial processing	Lane departure warning [11]
	Good at low-level computing	Complex to program	Pedestrian recognition [12]
GPGPU	Highly parallelizable	Power-hungry	Pedestrian detection [13]
	Programming flexibility	Complex to program	Road detection [14]
DSP	Well suited for image processing	Medium speed performance	Object detection [15,16]
	Good price to performance ratio	Complex to program	Lane departure warning [17]
μP	Best for high-level processing	Poor parallelization	Lane departure warning [18]
	Easy to program	High power consumption	Vehicle detection [19]

**Table 2:** Number of Operations per Clock Cycle per Multiprocessor.

Arithmetic Instructions	Throughput
16-bit floating-point add, multiply, multiply-add	Not Applicable
32-bit floating-point add, multiply, multiply-add	192
64-bit floating-point add, multiply, multiply-add	8
32-bit integer add, extended-precision add, subtract, extended-precision subtract	160
32-bit integer multiply, multiply-add, extended-precision multiply-add	32

**Table 3:** Memory types in GPGPU.

Memory Type	Lifetime	Place	Scope	Cache
Global memory	Application	Off-chip	All threads	L2 + L1
Local memory	Thread	Off-chip	Thread	L2 + L1
Constant memory	Application	Off-chip	All threads	Constant cache
Texture and surface memory	Application	Off-chip	All threads	Texture cache
Shared memory	Block	On-chip	Block	X
Register	Thread	On-chip	Thread	X

**Table 4:** FPGA resource utilization.

Core Name	BRAM 18K	DSP48	Flip-Flops	LUT
Update Prior & Motion Detection	256	18	14462	47311
UPdate Posterior	740	860	78172	91047

**Table 5:** Summary of final desings.

Platform	Aritmetic Cores	Caches	Membus Width	DDR
Tegra TK1 (32-bit version)	192 CUDA FP32/852 MHz	16-32-64 KB L1	64-bit	7 MB
		128 KB L2		
		64 KB Registers		
Enclustra ZX1	878 DSP	1.9 MB BRAM	32-bit	1 MB
(extrapolated to ZC7045)	INT48/176 MHz	550 B Registers		

**Table 6:** GPGPU errors after Posterior stage.

Frames	Velocity: Mean (km/h)	Velocity: Standard Deviation (km/h)	Angle: Mean (rad/s)	Angle: Standard Deviation (rad/s)
1–1013	0.0025	0.3194	$3.6383\times 10^{-5}$	0.005
1014–1535	6.2211	10.9009	0.0296	0.1986

**Table 7:** FPGA errors after Posterior stage.

Frames	Velocity: Mean (km/h)	Velocity: Standard Deviation (km/h)	Angle: Mean (rad/s)	Angle: Standard Deviation (rad/s)
1–1535	0.02376	0.46872	0.0043	0.1515

**Table 8:** GPGPU version, latency per block (ms).

Step	Curve Fitting	R-Square
Update Prior	1.253	-
Motion Detection	0.0854	-
Update Posterior	0.3028 + 0.005607x	0.9819

**Table 9:** FPGA version, latency per block (ms).

Step	Curve Fitting	R-Square
Update Prior	0.4245	-
Motion Detection	0.2025	-
Update Posterior	1.0816 + 0.0137x	0.7352
